# NAD^+^ metabolism, stemness, the immune response, and cancer

**DOI:** 10.1038/s41392-020-00354-w

**Published:** 2021-01-01

**Authors:** Lola E. Navas, Amancio Carnero

**Affiliations:** 1 Instituto de Biomedicina de Sevilla (IBIS), Hospital Universitario Virgen del Rocío, Universidad de Sevilla, Consejo Superior de Investigaciones Científicas, Sevilla, Spain; 2 CIBER de Cancer, Sevilla, Spain

**Keywords:** Cancer stem cells, Molecular medicine, Cancer metabolism, Cancer therapy

## Abstract

NAD^+^ was discovered during yeast fermentation, and since its discovery, its important roles in redox metabolism, aging, and longevity, the immune system and DNA repair have been highlighted. A deregulation of the NAD^+^ levels has been associated with metabolic diseases and aging-related diseases, including neurodegeneration, defective immune responses, and cancer. NAD^+^ acts as a cofactor through its interplay with NADH, playing an essential role in many enzymatic reactions of energy metabolism, such as glycolysis, oxidative phosphorylation, fatty acid oxidation, and the TCA cycle. NAD^+^ also plays a role in deacetylation by sirtuins and ADP ribosylation during DNA damage/repair by PARP proteins. Finally, different NAD hydrolase proteins also consume NAD^+^ while converting it into ADP-ribose or its cyclic counterpart. Some of these proteins, such as CD38, seem to be extensively involved in the immune response. Since NAD cannot be taken directly from food, NAD metabolism is essential, and NAMPT is the key enzyme recovering NAD from nicotinamide and generating most of the NAD cellular pools. Because of the complex network of pathways in which NAD^+^ is essential, the important role of NAD^+^ and its key generating enzyme, NAMPT, in cancer is understandable. In the present work, we review the role of NAD^+^ and NAMPT in the ways that they may influence cancer metabolism, the immune system, stemness, aging, and cancer. Finally, we review some ongoing research on therapeutic approaches.

## Introduction

Nicotinamide adenine dinucleotide (NAD^+^) is an abundant metabolite that plays an essential role in the maintenance of cellular homeostasis. NAD^+^ acts as a cofactor in multiple reduction-oxidation (redox) reactions related to energy production, glycolysis, the tricarboxylic acid (TCA) cycle, oxidative phosphorylation (OXPHOS), fatty acid oxidation (FAO), and serine biosynthesis.^1–3^ On the other hand, NAD^+^ is a substrate for different signaling enzymes, such as sirtuins, PARPs, and cADPRSs. In these reactions, NAD^+^ is degraded and cleaved into ADP ribose and nicotinamide (NAM), which can be recycled.

**Fig. 1:**
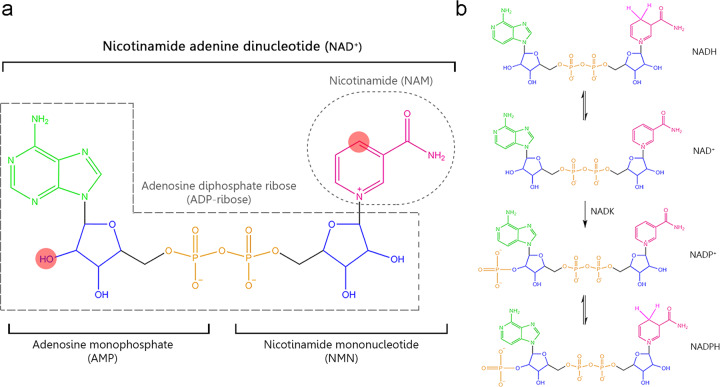
**a** Structure of nicotinamide adenine dinucleotide (NAD^+^). This molecule is formed by adenosine monophosphate (AMP) linking to nicotinamide mononucleotide (NMN). AMP is formed by adenosine (in green), ribose ring (in blue) whose hydroxyl (marked in red) can be phosphorylated resulting in NADP+, and phosphate group (in orange). NMN is formed by a ribose ring, phosphate group, and nicotinamide (NAM) (in pink) that is responsible for redox NAD^+^ functions, thus, the atom carbon (marked in red) is capable of accepting a hydride anion (H+, 2e-) leading to the reduced form NADH. In addition to the classical redox functions, NAD^+^ acts as a substrate of multiples enzymes donating the group adenosine diphosphate ribose (ADP-ribose) and releasing nicotinamide as the catabolic reaction product. **b** Structure of the different states of NAD^+^. NAD^+^ can be phosphorylated to NADP^+^ by NADK enzyme. Thus, NAD^+^ and NADP^+^ can be reduced to NADH or NADPH, respectively. NADH nicotinamide adenine dinucleotide, NAD^+^ nicotinamide adenine dinucleotide, NADP^+^ nicotinamide adenine dinucleotide phosphate, NADPH reduced nicotinamide adenine dinucleotide phosphate

**Fig. 2:**
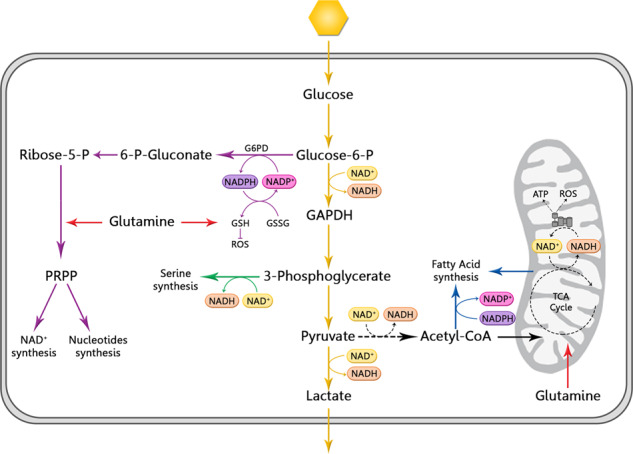
NAD^+^ in cancer metabolism. Cancer cells rely on glycolysis (pathway in yellow) than mitochondrial oxidative phosphorylation (OXPHOS) (in black) what is called the Warburg Effect. The pentose phosphate pathway (in purple), serine synthesis (in green), and fatty acid synthesis (in blue) are also upregulated in cancer and depend on glycolysis as the core of cancer metabolism. Glutaminolysis (in red) is also upregulated as the main nitrogen source. All these pathways require the cofactor NAD^+^/NADH/NADP+/NADPH as essential cofactors to support faster energy production, counteract ROS production, and synthesis building blocks for tumor proliferation. G6PD glucose-6-phosphate dehydrogenase, GSH glutathione, GSSG glutathione disulfide, ROS reactive oxygen species, GAPDH glyceraldehyde 3-phosphate, LDH lactate dehydrogenase, PRPP 5-phosphoribosyl-1-pyrophosphate

**Fig. 3:**
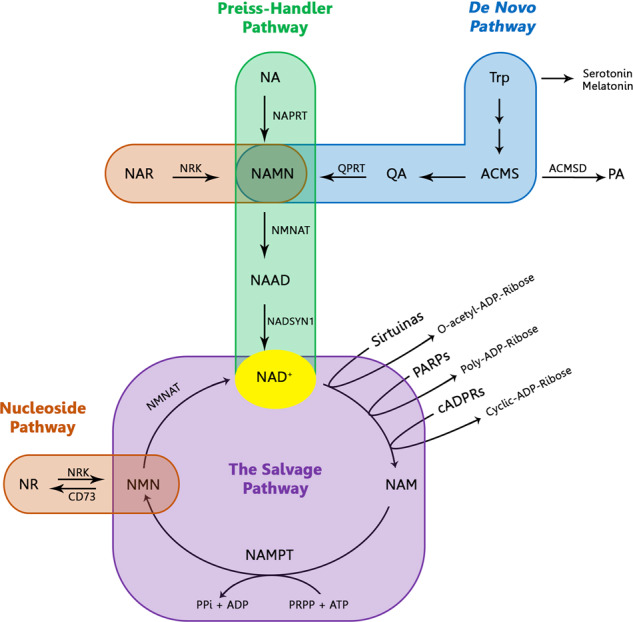
NAD^+^ biosynthesis pathways. NAD^+^ can be synthesized from the different dietary precursors by de novo pathway (from Trp), the Preiss–Handler pathway (from NA), and the nucleoside pathway (from nicotinic NAR or NR). However, the main source of NAD^+^ is the salvage pathway where the catabolic product (NAM), from NAD^+^-consumed enzymes (sirtuins, PARPs and cADPRSs), is recycled to reconstitute NAD^+^

To respond to the high and dynamic NAD^+^ cellular demand for both redox and nonredox processes, mammalian cells must synthesize NAD^+^ from different dietary precursors through the *de novo* pathway and the Preiss–Handler pathway (Fig. 1). Indeed, a chronic dietary deficiency of these precursors leads to pellagra, which is characterized by diarrhea, dermatitis, dementia, neurological damage, and even death. During an investigation into a treatment for pellagra in the 1920s, NAD^+^ was discovered as an essential metabolite for cells^2,4,5^ (Fig. 2). Alternatively, cells can be recycled and NAD^+^ reconstituted from its catabolic products via the salvage pathway, which is the preferential route for cells to replenish NAD pools^6–10^ (Fig. 3). NAD levels are limiting components, and the availability of NAD is essential for cellular functionality. Therefore, the biosynthesis, subcellular localization, and systemic transport of NAD and its intermediates are key to the regulation of various biological processes with significant impact on cellular and tissue functionality. To date, studies have revealed a complex layer of tissue/organ-specific effects and differential roles for various NAD intermediates.

## NAD^+^ concentration and compartmentalization

Although the oxidized form, NAD^+^, is the most abundant and ubiquitous state in cells, its availability may fluctuate. Indeed, the free NAD^+^ concentration largely varies depending on several factors, including its subcellular compartment; the cellular type; glucose level, including deprivation; caloric intake and restriction; exercise; age; and circadian cycles. It has been shown that the cytosolic and nuclear NAD^+^ pool concentrations are quite similar (approximately 100 µM) because these compartments are interconnected. The mitochondrial NAD^+^ pool, with a higher concentration (~250–500 µM), is an independent source since the mitochondrial membrane is impermeable to NAD^+^ and NADH. The free NAD^+^ concentration also depends on the cell type. For example, myocytes have a greater mitochondrial NAD^+^ pool, whereas hepatocytes and astrocytes have greater cytosolic NAD^+^ pools.^11^

In addition, NAD^+^ concentration may oscillate throughout the day due to circadian regulation. The CLOCK:BMAL1 complex activates the promoter of NAMPT, the critical enzyme of the NAD^+^ salvage pathway, in a cyclic manner every 24 hours, increasing NAD^+^ production. In addition, SIRT1, an NAD^+^-consumed enzyme, counteracts the effect of the circadian complex by deacetylating BMAL1 and the histones in the promoter region of other regulated genes.^12–14^

NAD^+^ has a short half-life, ~1 h in mammalian cells,^15^ and limited ability to diffuse through cell membrane barriers.^16^ This impermeability ensures rapid local regulation of NAD-dependent physiological processes and extensive compartmentalization of many enzymes involved in the biosynthesis or consumption of NAD^+^ has also been described. Therefore, the concentration of NAD^+^ is not uniform within a cell. Most bioenergy- and NAD^+^-dependent signaling pathways are activated in different subcellular compartments, and locally accessible NAD^+^ is required for these metabolic processes.^17,18^ NAD^+^ has been detected in the nucleus and in almost all cytoplasmic organelles.^19–21^

The extracellular NAD^+^ concentration in the blood serum of mammals is maintained at a low level, between 0.1 and 0.5 μM, under physiological conditions.^22^ However, the extracellular NAD^+^ level is connected to the intracellular NAD^+^ pools. Cells, especially cancer cells, release intracellular NAD^+^ into the culture medium, indicating that NAD^+^ may serve as an autocrine or paracrine signaling molecule for nearby cells.^23^ Extracellular NAD^+^ restores intracellular NAD^+^ pools and helps counteract cell the death induced by NAMPT inhibition.^7,8,24,25^

Extracellular NAD^+^ is mainly consumed through the action of CD38, a ubiquitously expressed transmembrane NAD^+^ glycohydrolase.^23,26^ CD38 expression delays cancer development by lowering NAD^+^ levels, with a reduction in cell metabolism that leads to cell cycle arrest.^27^ Extracellular NAD^+^ also plays important roles in immune modulation. Inflammation can trigger the release of intracellular NAD^+^ into the extracellular space, which in turn fine-tunes the immune response.^28^ Specifically, extracellular NAD^+^ acts as a proinflammatory cytokine that stimulates granulocytes, augmenting chemotaxis.^29,30^ In addition, evidence suggests that extracellular NAD^+^ has an important regulatory role in the antitumor T-cell response. Foxp3+ regulatory T cells (Tregs) are specifically susceptible to NAD^+^-induced cell death, and systemic administration of NAD^+^ selectively depletes Tregs, promoting antitumor responses in xenograft tumor mouse models.^31^ T cells with reduced surface expression of CD38 exhibited higher levels of NAD^+^ and superior antitumor responses, including enhanced oxidative phosphorylation, higher level of glutaminolysis, and altered mitochondrial dynamics.^32^

## Redox functions of NAD^+^

When initially discovered, the function of NAD^+^ was attributed to the universal electron transporter in redox reactions for obtaining energy in catabolic processes. During glycolysis, NAD^+^ is reduced to NADH upon the oxidation of glyceraldehyde 3-phosphate (GAPDH) in the cytosol. Each glucose molecule generates 2 molecules of NADH, which can be shuttled into the mitochondria; 2 molecules of ATP; and 2 molecules of pyruvate. Under anaerobic conditions, pyruvate can be reduced to lactate by lactate dehydrogenase (LDH), while NADH is oxidized to regenerate NAD^+^. When oxygen is available, pyruvate can be oxidized to acetyl CoA, with a reduction of NAD^+^.^1,2,33^ Another source of acetyl CoA is generated by fatty acid beta-oxidation (FAO), where NAD^+^ is reduced to NADH.^34^

In mitochondria, acetyl CoA enters the TCA cycle, generating 8 molecules of NADH. All these NADH molecules and those generated from glycolysis can be oxidized back to NAD^+^ in complex I, called NADH/coenzyme Q reductase, of the electron transport chain (ETC). Ultimately, 30 molecules of ATP are produced from these NADH molecules; however, a substantial number of reactive oxygen species (ROS) are also generated.^1,2,33^ Excessive ROS levels may cause serious oxidative damage, which can be reduced by an antioxidant system formed by SODs, catalases, MAPKS, SIRTs, GSH, vitamin C and E.^35,36^

The production of ATP in the mitochondria and the maintenance of the membrane potential require NAD^+^ as a universal cofactor. NAD^+^ is reduced to NADH by gaining two electrons and a proton from substrates at multiple steps in the TCA cycle. Mitochondrial NADH is oxidized by donating its electrons to complex I (NADH:ubiquinone oxidoreductase) in the electron-transporter channel. These electrons are sequentially relayed from complex I to ubiquinone (coenzyme Q10), complex III, cytochrome c, and complex IV (cytochrome c oxidase), resulting in the reduction of oxygen to water. This flow of electrons is coupled to the pumping of protons by complexes I, III, and IV across the inner membrane and into the inner mitochondrial membrane space, generating a proton gradient. Protons re-enter the matrix through F0F1-ATP synthase, creating a flow that drives ATP synthesis. Because the TCA cycle and the electron-transporter channel require NAD^+^ and NADH, respectively, an optimal NAD/NADH ratio is needed for efficient mitochondrial metabolism. The mitochondrial NAD^+^ pool is relatively distinct from that of the rest of the cell. Cytoplasmic NAD/NADH ratios range between 60 and 700 in a typical eukaryotic cell; however, mitochondrial NAD/NADH ratios are maintained at much lower levels. Numerous studies have conducted in-depth investigations into the factors that influence the mitochondrial NAD/NADH ratio.^37–40^

In contrast to NAD^+^, the phosphorylated form, NADP^+^, is required for the anabolism process, including for nucleic acid synthesis by the pentose phosphate pathway (PPP).^5,41^ In addition, its reduced form, NADPH, is essential for diminishing ROS levels. In fact, many antioxidant proteins depend on NADPH as a cofactor. For example, the glutathione disulfide (GSSG) reduction to glutathione (GSH) is driven by the NADPH generated during the first step of the PPP pathway, when glucose-6-phosphate is converted to 6-P-gluconate.^5^ NADPH is also an important cofactor in fatty acid synthesis (FAS), which is required for membrane synthesis in proliferating cells, using acetyl CoA as a precursor.^2^

The maintenance of intracellular redox homeostasis is a critical cellular process requiring a balance between reactive oxygen species (ROS) generation and elimination, as excessive levels of ROS can result in cell death.^42–45^ NAD^+^ is an important regulator of cellular ROS levels that can accumulate upon depletion of NAD^+^. This regulation is particularly important in cancer cells, which generally have increased ROS production and require very tight control of the ROS balance.^43–45^

NAD has been shown to contribute to the cellular capacity to tolerate oxidative stress in a number of studies. In CRC or glioma cell lines, NAMPT functions to increase NADH pools, protecting cells against oxidative stress.^6–10^ In breast cancer models, the increased of NAD^+^ pool can be converted to NADPH through the pentose phosphate pathway, thus maintaining glutathione in the reduced state. In addition, in several studies, NAD depletion enhanced cancer cell susceptibility to oxidative stress through a reduction in antioxidative capacity by downregulating antioxidant proteins.^6–10,43–46^ In many of these studies, a possible therapeutic window was observed upon NAMPT inhibition, since the response of the normal cells was different from that of the tumor cells. However, this response to NAMPT inhibition is not a universal feature of tumor cells; that is, not all cancer cells exhibit an increase in ROS or NAD^+^ depletion upon NAMPT inhibition. In Ewing sarcoma and some NSCLC cell lines, for example, inhibiting NAMPT does not create an imbalance in ROS, suggesting compensatory mechanisms maintain NAD^+^ levels.^47–49^ However, several studies have reported the efficacy of combining NAMPT inhibitors with ROS-inducing agents, resulting in excessive ROS production, which enhanced the efficacy of treatment against the growth of tumors.^6–10^

NAD availability in the cytoplasm is a key determinant of the rate of NADH and pyruvate flux to mitochondria and, therefore, the rate of glycolysis. Eliminating cytoplasmic NAD^+^ blocks glycolysis and leads to cell death.^3,50–54^ However, many works have suggested the NAD^+^ biosynthetic machinery inside the mitochondria maintain the mitochondrial NAD^+^ pool in response to environmental and nutritional stresses.^3,17^

Important metabolic and molecular insights into the essential metabolic role of NAD^+^ in redox function were gained from studies on NAMPT-KO mice.^55^ The authors reported that NAMPT-null (NAMPT^−/−^) mice showed embryonic lethality at early stages, and the adult mice die within 5–10 days after NAMPT depletion. NAMPT-null mice have diminished NAD^+^ synthesis and no other NAD biosynthetic enzymes. These defects lead to a profound dysregulation of other NAD-dependent genes, such as SIRTs, PARPs, and acetylated proteins in the Sirt3-Foxo4 axis, and suppression of the TCA cycle. This TCA cycle suppression results in a large decrease in ATP, which adversely affects all ATP-dependent proteins, including those involved in nutrient transport, mobilization, and uptake, leading to mouse death.^55^ However, other roles of nonredox function on this phenotype were not specifically explored.

## Nonredox functions: NAD^+^ as a substrate

Both NAD^+^/NADH can be converted in an almost unlimited way without being consumed.^56^ Therefore, in theory, there should be the amount of NAD^+^ should equal the amount of NADH in cells. The reality, however, is quite different. NAD^+^ is ~600–1100-fold more abundant than NADH,^4^ because NAD^+^ participates in other multiple molecular nonredox processes, including DNA repair, cell signaling, posttranslational modifications, mitochondrial metabolism, inflammatory responses, senescence, and apoptosis.^1,5,11,30,57–63^ In these nonredox reactions, NAD^+^ is consumed as a substrate by different types of enzymes, including sirtuins, PARPs, and the cADPRS family of ectoenzymes. These enzymes use NAD^+^ as a donator of ADP-ribose and release NAM as a recycling reaction product.

PARPs, located in the nucleus, mediate many biological processes, including DNA repair, transcriptional regulation, and metabolism (41, 45), by transferring long chains and branches of ADP-ribosyl polymers to their protein substrates. CD38 and CD157 from the cADPRS family of ectoenzymes generate cyclic ADP-ribose (cADPR) and NAADP, potent second messengers for calcium signaling.^30^ In the sirtuin family of proteins, NAD mediates deacetylation, demalonylation/desuccinylation, and ADP-ribosylation of target proteins.^18,64–68^ The PARP and cADPRS families significantly affect cellular NAD levels,^30^ limiting NAD availability for other NAD-consuming enzymes. Thus, it has been shown that the inhibition of PARP1 or CD38 increases NAD^+^ levels, leading to an increase in the activity of SIRT1.^69^ Therefore, these NAD^+^-dependent enzymes, namely, sirtuins, PARPs, and cADPRSs, are able to coregulate each other in competition for the same intracellular limiting NAD^+^ pool. For example, SIRT1 can inhibit PARP1 by deacetylation, increasing NAD^+^ levels, whereas PARP2 represses the transcription of sirtuins.^4,59,70^ The importance of the NAD^+^ role in nonredox reactions has been reestablished in recent years, and consequently, NAD^+^ has been proposed as a link between metabolism and signaling in many physiological processes and diseases.^71^

## NAD^+^ in pluripotent and stem cells

Somatic cells can acquire self-renewal capacity and other stem properties through dedifferentiation. The expression of some transcription factors, such as Yamanaka factors (OSKM), including Oct3/4, Sox2, c-Myc, and Klf4, reprograms somatic cells to induced pluripotent stem cells (iPSCs).^72^ This pluripotency reprogramming process is closely related to cancer cell dedifferentiation to cancer stem-like cells (CSCs).^73^ Similar to pluripotent and stem cells, CSCs have the capacity to self-renew and differentiate into progenitor cells. Differentiated cancer cells are able to acquire stem-like properties through dedifferentiation as can iPSCs.^74–77^ Both CSCs and iPSCs activate the Warburg effect, enhanced in a hypoxic state, to maintain the stemness phenotype. Therefore, the term glycolysis is used to describe stemness.^74,77,78^

In this context, a high NAD^+^/NADH ratio has been found in human iPSCs and embryonic stem cells (ESCs).^78^ Son et al. proposed NAD^+^ as a modulator for stem cell pluripotency in iPSCs through OSKM factor promotion. They also showed that NAM supplementation bypasses senescence and apoptosis and consequently promotes reprogramming during iPSC generation.^79^ In fact, an analysis of the iPSC and stem cell transcriptomes showed highly upregulated levels of NAMPT.^46^

NAMPT and/or NAD reduces the pool and inhibits the proliferation of neural progenitor stem cells.^80^ In many lineages, NAD and NAMPT may be necessary not only for proliferation but also for cell fate decisions. Neural stem progenitor cells require NAMPT for cell cycle progression. However, in the oligodendrocyte lineage, NAMPT downregulation reduced oligodendrogenesis in vivo.^81^

NAMPT inhibition strongly decreases the levels of OCT4 and other pluripotency factors, such as Sox2, Klf4, or NaGOG, and EMT factors, such as Twist, Snai1, Slug, or FoxC2.^7,82^ Downregulation of NAMPT also increases the expression of pluripotent differentiating factors. It has been shown that, in human stem cells, a 50% reduction in NAD^+^ level causes spontaneous differentiation and apoptosis.^79^ IPs have higher levels of NAD synthesis salvage pathway enzymes and are dependent on them for survival.^79^ Moreover, nicotinamide, the precursor of NAD, increases the survival of hSCs.^83^ Supraphysiological NA levels increase the extent of IP reprogramming.^79^ In mouse models in vivo, an intracellular NAD+ level boost by NA riboside treatment rejuvenated intestinal, neural and muscle stem cells and extended the lifespan.^84,85^ Dietary supplementation with NAD precursors also increases mitochondrial activity.^84^ Therefore, increasing NAD in the cell supports stemness and pluripotency, in which efficient mitochondrial function seems to be necessary.^85^

However, NAD is not only essential for metabolism, but also in other activities such as building blocks of other cellular synthetic pathways, DNA repair, signal transduction molecule, autophagy, or epigenetic regulation. Studies have shown that NAD is an important cofactor for neural functions that have high energetic demands. The increase in NAD^+^ levels delays senescence in mesenchymal stem cells.^81^ NAD^+^ and NAMPT levels in neural tissues are reduced with age, and NAD replenishment reduces the severity of ataxia telangiectasia neuropathology, restoring neuromuscular function and memory loss that accompany aging processes.^86–88^ NAD stimulates DNA repair through PARP, improving mitochondrial activity.

In parallel, an increase in NAMPT activity boosts autophagy,^86,89^ while a decrease in OCT4 enhances autophagy inhibition. These findings have led to the hypothesis that basal levels of autophagy are required for the pluripotency acquired by CSCs.^82^ Autophagy induction by rapamycin, upon serum starvation or through a BECNI peptide showed an increase in stemness properties. NAMPT, through OCT4, regulates mTOR phosphorylation, an important negative regulator of autophagy. In normal muscle stem cells, basal levels of autophagy contribute to the maintenance of stemness and pluripotency.^90–93^

Therefore, an increase in NAD^+^ led to increased stem cell proliferation and pluripotency, as determined by SC marker expression.^94^ However, NAD increases also led to reduced activation of the WNT canonical pathway. In addition, when treated with NAD^+^, embryonic SCs became resistant to differentiation induced by BMP4. Furthermore, NAD^+^ failed to induce stemness morphology or activate stem genes, such as nanog, GBX2, REX1, or OTX2. This suggests that NAD alone is not able to induce a full stem transcriptional network^94^ but is able to induce an intermediate SC state that might be positively affected by additional reprogramming signals. These primed SCs convert most glucose to lactate, facilitating the recycling of NAD from the cytosol and ensuring their utilization in other cellular synthesis pathways. NAD^+^ also enhances mitochondrial suppression of oxygen and ATP production.^94^ Naive embryonic stem cells, such as those primed by NAD, also consume glutamine as an important nutrient and produce a-ketoglutarate for H3K27me3 demethylation, thereby maintaining the hypomethylated state of embryonic stem cells.^95–97^

## NAD^+^ and aging

An imbalance in the NAD^+^/NADH or NADP^+^/NADPH ratio can destabilize cellular homeostasis, causing not only pellagra but also aging, neurodegenerative diseases, inflammatory alterations, infections, cardiovascular diseases, and cancer.^98,99^ The most studied context for NAD^+^ depletion is aging, in which a significant reduction in sirtuin activity and its reported anti-aging effects have been observed^100–102^ and references therein). NAD^+^ depletion, as previously described, causes mitochondrial dysfunction, a decline in energy production and accumulated ROS that produce high oxidative stress. The increase in ROS and oxidative stress combined with an accumulation of genome mutations leads to chronic PARP activation. High PARP levels lead to the rapid consumption of the NAD^+^ pool and, consequently, to a decrease in sirtuin levels, promoting aging.^59,70,103,104^ In contrast, treatment with NAD^+^ precursors, as well as other forms of energy consumption, such as through exercise, caloric restriction, fasting, and low glucose availability, can counteract aging effects by upregulating NAMPT, which can reconstitute NAD^+^ levels and promote sirtuin activation.^5^

Many of these aging hallmarks are commonly manifest with cancer hallmarks.^98^ It is not surprising that aging and cancer are two linked processes. Gomes et al. suggested that ROS production increases due to NAD^+^ reduction during aging and promotes hypoxia-inducible factor-1α (HIF-1α) accumulation, leading to metabolic reprogramming, which is a hallmark of cancer.^105^ On the other hand, the recovery of NAD^+^ levels by supplementation with nicotinamide riboside, NR, precursor of the nucleoside pathway, enhances the function of mitochondria and stem cells in elderly mice.^84,106^ Consistently, the overexpression of NNMT and NMNAT3 and subsequent restoration of the mitochondrial NAD^+^ pool increases the lifespan of human MSCs by inhibiting cell senescence and increasing the reprogramming efficiency of differentiated somatic cells.^107^

Aging is characterized by a decline in the regenerative potential of tissues. Reduced stem cell function leads to the diminished replenishment of the tissue in essentially all adult stem cell compartments.^108–110^ The lack of efficient repair of these damaged tissues is the consequence of stem cell decline and aging.^111,112^ The importance of NAD^+^ for maintaining the pool and pluripotency of stem cells makes it an essential cofactor during aging.

A reduction in NAD^+^ synthesis is one of the main causes of NAD depletion in aging. In accordance, NAMPT levels decrease in many tissues during organismal aging.^113–115^ Although several factors seem to affect NAMPT levels, it has been reported that the CLOCK:BMAL1 complex, the core transcription factor in circadian rhythms, binds directly to the regulatory region of NAMPT and regulates its expression.^12,46^ NAD^+^ and NAMPT levels show circadian oscillations amplified by the loop consisting of NAMPT-NAD-SIRT1, since SIRT1 regulates circadian gene expression by deacetylating BMAL1 and PER2.^116,117^ Therefore, aging decreases the circadian rhythms that reduce NAMPT and therefore NAD, and in turn, these changes regulate aging.^118^

During aging, ROS levels increase, causing an increase in DNA damage.^62^ Therefore, there is an increase in PARP activity and the amount of NAD used as a substrate.^119,120^ Genetic or pharmacological inhibition of PARP1 prevents NAD decline during aging.^121^ However, PARP inhibition impedes DNA repair, increases DNA damage, and accelerates cancer initiation.

Aging causes multiple changes in body metabolism and vice versa, tightly connecting both phenotypes. Increases in obesity and changes in fat accumulation are associated with aging, and obesity also accelerates aging by inducing inflammation in several tissues.^122^ During aging, NAD levels decrease in various tissues, including liver and adipose tissues.^123,124^ Therefore, all the pathways and metabolic reactions in which NAD is involved are also altered by aging, and this decreased metabolic activity has been associated with numerous aging-related metabolic diseases, such as diabetes.^86,125^ NAD levels are also related to insulin secretion and sensitivity. In fact, mice with reduced levels of NAMPT, such as genetic NAMPT heterozygous mice, showed low glucose tolerance due to impaired insulin secretion.^126,127^ The administration of the product of NAMPT, nicotinamide mononucleotide, NMN, led to recovery of both NAD levels and insulin production. Interestingly, adipocyte-specific NAMPT-depleted mice also showed insulin resistance in their muscle and liver.^128^ Adipocytes and other cells can secrete eNAMPT, and plasma levels of eNAMPT are usually increased in obesity. Obesity also reduces NAMPT by inducing the expression of miR34a. In contrast, reducing miRNA34a levels increases the NAMPT enzyme and restores NAD levels.^129,130^

Nutritional supplementation of NAD precursors may reduce insulin resistance during obesity and aging^80,102^ and references therein). This effect seems to involve the sirtuin pathway. NR administration increases NAD^+^ levels, facilitating the deacetylation of FOXO1 by upregulating SIRT1.^131^ In addition, the increase in mitochondrial NAD levels by exogenous NR also promotes the deacetylation of SOD2 and NDUFA9 through the increased levels of SIRT3.^131^ Exogenous NAMPT product NMN supplementation also increases the NAD pool, ameliorating liver insulin resistance. In addition, NMN supplementation restores the expression of genes involved in oxidative stress, the inflammatory response, and circadian rhythms.^114,132^ Sirtuins have been considered major targets of NAD regulation of aging and longevity. It is now clear that NAD and/or related metabolites have other roles in the regulation of aging metabolism that are independent of sirtuin activity.^80,102^

Very interestingly, CD38-KO mice showed increased longevity and were protected against NAD level decreases associated with age, indicating that other NAD-consuming enzymes may play an important role in age- and age-dependent diseases.^133^

## NAD^+^ and NAMPT in inflammation and the immune microenvironment

### Innate and adaptative responses in cancer

Hematopoiesis declines with age, resulting in a diminished production of adaptive immune cells—a process termed immunosenescence—.^134^ Aged mice show a decrease in the reconstitution capability of their hematopoietic stem cells (HSCs), correlating with an increase in DNA damage^135,136^ and with an increase in the levels of senescence effectors, such as p16INK4a.^137^ In fact, old hematopoietic stem cells from INK4a^−/−^ (KO) mice showed better engraftment capacity compared with the HSCs from old wild-type mice.^137^ Indeed, old wild-type mice show higher number of phenotypically identified HSCs but their “stem capacity” is diminished.

In addition to tumor cells, the microenvironment contains a myriad of nonneoplastic components that can support, or inhibit, depending on the conditions in each case. The possible metabolic modifications of all cellular components or the microenvironment affecting the metabolism, transcriptome, properties, and phenotype of the tumor have been clearly described. NAD metabolism and its responses are based on cross talk between cancer and infiltrating immune cells, affecting tumor growth, malignity, and immune evasion.^138–140^ The regulation of immune responses in the tumor microenvironment depends on different types of host cells, including endothelial cells, mesenchymal stem/stromal cells, tumor-associated fibroblasts (TAFs and CAFs), and tumor-infiltrating immune cells, such as lymphocytes, macrophages, myeloid suppressor cells, and tumor-associated neutrophils.

TAFs interact with stromal cells by secreting growth factors, cytokines, and chemokines, including IL6, TGFβ, and CCL2, to amplify immune evasion systems, attracting immunosuppressive cells into the tumor microenvironment, and they also form a network of proinflammatory mediators at the tumor site. Tumor-infiltrating lymphocytes (TILs) are essential for the correct immune response. The composition of tumor-infiltrating lymphocytes depends on many factors, including tumor type, localization, stage, stemness, and response to previous therapy. However, the presence of lymphocytes inside the tumor is often associated with a better response to immunotherapy based on immune checkpoints.^138^ However, excessive or chronic inflammation exhausts immune cells due to the constant exposure of T antigens, causing dysfunction with the maintained expression of inhibitory receptors such as PD1 and CTLA4, which alter transcription. Tumor-associated macrophages are important mediators of tumorigenesis and are also form reciprocal loops with CAFs. These fibroblasts promote monocyte recruitment and polarization to the M2 phenotype. On the other hand, M2 macrophages induce CAF activation. Tumor-associated macrophages induce immune suppression,^138,141^ mediating the sustained expression of inhibitory receptors. TAL also releases several cytokines, such as IL10 and TGFβ, contributing to the immunosuppressive environment.

TAMs and MSDCs are usually considered key for tumor immunosurveillance evasion;^142^ however, myeloid suppressor cells and tumor-associated neutrophils have recently attracted great attention in the field. The subpopulations of myeloid cells defined by their immunosuppression capacity are generated because of aberrant myeloid cells recruitment in tumors. During tumorigenesis, CXCR4/CXCL12 mobilizes these cells to infiltrate tumors and promote tumorigenesis. These myeloid immunosuppressive cells disrupt major mechanisms of immunosurveillance, including antigen presentation by dendritic cells, T-cell activation, and/or the cytotoxicity of NK cells.^143^ On the other hand, attracted by the secretion of different cytokines, TANs migrate to the tumor, where they promote a tumorigenic phenotype, supporting tumor growth and suppressing immune responses.^144^

Many studies have shown that NAMPT can be regulated by different inflammatory signals in different types of immune cells.^138^ Lipopolysaccharides from pathogens are stimuli for inflammatory TNFα, IL6, or leptin activation, which leads to the upregulation of NAMPT transcription in several immune cell types.^145–147^ Interestingly, extracellular eNAMPT increases the release of inflammatory cytokines.^148^ eNAMPT increases the levels in and release of IL6, IL10, TNFα, and IL1b from PBMCs and CD14+ monocytes. In response to eNAMPT, other stimulatory molecules, such as CD40 or CD80, are also upregulated through the activation of several intracellular pathways, namely, PI3K, MEK, p38, and JUN.^149^ eNAMPT induces IL6 secretion, which activates the autocrine/paracrine loop mediated by STAT3, increasing the resistance of macrophages to the cell death induced by endoplasmic reticulum stress.^46^ Interestingly, some properties of CSCs are enhanced through a mechanism that is independent of nicotinamide phosphoribosyltransferase enzymatic activity.

NAMPT regulates the metabolic adaptation of myeloid cells. Metabolic reprogramming of immune cells is required for both proinflammatory and anti-inflammatory responses. In general, the proinflammatory M1 phenotype manifests in glucose uptake and glycolysis,^150^ while the anti-inflammatory M2 phenotype manifest in OXPHOS and the TCA cycle activity.^151^ However, M2 macrophages can use glycolysis if OXPHOS is disrupted.^152^ In M1 macrophages, PPP-generated NADPH is essential since ROS production is maintained by NADPH oxidase.^153^ Both intra- and extracellular NAMPT may regulate the processes of macrophage differentiation, polarization, and migration.^138^ NAMPT has an important role in the tumorigenic microenvironment in leukemia, with eNAMPT playing an important role in macrophage differentiation to the M2 phenotype and polarization in tumorigenesis-associated macrophages.^140^ Intracellular NAMPT can also inhibit CXCR4 transcription through NAD/sirtuins, increasing the production of nitric oxide through the mobilization of MDSCs.^154^

As a consequence of NAMPT regulation, different levels of NAD are typical of each state of macrophage differentiation. High NAD levels are detected, in general, in the M1, proinflammatory, macrophages, while in M2, anti-inflammatory, macrophages, NAD levels are usually lower. In M1 macrophages, high levels of cytosolic NAD seem to be necessary for the efficient activation of myeloid function.^155^ A NAD^+^ increase also seems to be necessary for the metabolic reprogramming of the transition from early inflammation, based mainly on glycolysis, to acute inflammation, based on catabolic energy and fatty acid oxidation metabolism.^156,157^ During these processes, Sirt1/6 deacetylases also regulate metabolism, reducing glycolysis and increasing fatty acid oxidation coupling metabolism with the inflammatory response. Furthermore, in monocytes, TNFα induces BAMPT via HIF1α, and NAMPT further induces IL6 and TNFα transcription, regulating myeloid cell activation in a feedback loop.^158^

G-CSF, in contrast to GM-CSF, upregulates NAMPT, triggering NAD/SIRT1 granulopoiesis via C/EBPα/β upregulation.^159^ Pharmacological NAMPT inhibition increased the levels of C/EBPα acetylated and reduced the levels of expression of its target genes, such as G-CSF and G-CSFR. Furthermore, ectopic upregulation of NAMPT in a myeloid leukemia cell line triggered myeloid differentiation.^160^ Extracellular eNAMPT has cytokine functions acting through TLR4, and in human lung endothelial cells, eNAMPT activates an inflammatory response through the NFκB signaling pathway.^161^

During aging, diverse cellular and environmental stresses are known to cause cellular senescence and chronic inflammation, both of which stimulate the release multiple inflammatory cytokines, such as IL1β, IL6, and TNF, promoting further tissue damage.^162^ These inflammatory cytokines reduce the expression of NAMPT.^163^ Moreover, TNFα and IL1β inhibit the circadian rhythm CLOCK:BMAL1 complex.^164^ Therefore, inflammation appears to suppress NAMPT, decreasing NAD levels during aging in multiple ways.^102^ Several works have reported that the levels of eNAMPT, mostly in adipose tissue but also in plasma, correlate with the levels of inflammatory cytokines.^165^ Interestingly, the 5′ promoter region of the human CD38 gene contains binding sites for several cytokines, such as TNFα, NF-IL6, IL13, and IL4.^166,167^ TNFα treatment promotes the expression of CD38 ^168^ Thus, an increase in inflammation or senescence through inflammatory cytokines may induce CD38 expression during aging. Indeed, it has been observed that CD38 levels increase significantly during aging, with an increased consumption of NAD contributing to NAD reduction during aging.^123^

### CD38 and T-cell activation

CD38 is widely expressed in immune cell types and nonhematopoietic cells,^169^ and high levels are expressed in several hematopoietic malignancies. CD38 degrades circulating NAD and its precursors NMN and NR, preventing NAD synthesis.^123^ CD38 and NAD regulate T-cell phenotypes and responses.^138^ Expressed by immune and tumor cells, CD38 is involved in the activation and mobilization of intracellular Ca2+ signaling through the cADPR/NAADP metabolite product. CD38 metabolizes NAD, releasing NAM, allowing the continuous regeneration of NAD through NAMPT. These reactions in immune cells affect sirtuin activity by modifying the NAD pools. Therefore, the NAD/CD38/sirtuin axis regulates the transcriptome, metabolism, and cell fate of immune T cells. CD38 inhibitors were sufficient to reestablish T-cell proliferation, antitumor cytokine secretion and killing capability^138^ and references therein).

### Sirtuins in the immune response

NAD regulates the inflammatory response in immune and non-immune cells through Sirtuins.^170^ Epigenetic regulation of histones and non-histone proteins is induced by sirtuins and is essential for the development, reprogramming, and differentiation of the immune system and its related pathologies. Furthermore, numerous signal transduction proteins (such as receptors and cytokines) also regulate the transcriptional landscape through the epigenome, resulting in the ultimate regulation of the immune response.^171^ In myeloid cells, Sirt1 downregulates the NFκB pathway, decreasing inflammation and promoting metabolic reprogramming through PGC1α/AMPK and HIF1α stabilization. Sirt1 also induces T-cell tolerance by regulating the plasticity of helper T cells and Tregs.

## NAD^+^ and cancer

### NAD^+^ in cancer metabolism

Cancer cells show the ability to reprogram their metabolism to support a high proliferation rate and biomass production, even with limited energy resources and other adverse conditions. This Darwinian advantage enables tumor progression, development, and survival.^172^ Higher NAD^+^/NADH and NADP^+^/NADPH ratios are found in cancer cells compared to noncancerous cells, suggesting the important role of NAD^+^ in this metabolic conversion.^173^ Therefore, malignant cells shift from undertaking mitochondrial oxidative phosphorylation (OXPHOS) to invoking the glycolysis pathway, even under normal oxygen conditions with full and proper mitochondrial functioning, creating a pseudohypoxic state known as the Warburg effect.^174,175^

Several oncogenic mechanisms, such as the transcription factor HIF-1α, PI3K/Akt/mTOR pathway and c-Myc, upregulate genes encoding glucose transporters and glycolytic proteins, including GLUT1, GLUT3, and lactate dehydrogenase (LDH).^105,176^ Thus, cancer cells have significantly increased glucose uptake, which is the main source of energy supporting their uncontrolled proliferation.^174,175^ In this context, NAD^+^ levels must be increased to support the high glycolytic demand since two steps, GAPDH conversion and lactate production, depend on this molecule. Although the OXPHOS pathway is more energy efficient, since it generates 36 moles of ATP, cancer cells rely on glycolysis, which produces less ATP faster: 2 molecules of ATP are produced from 1 molecule of glucose.^3,176^ The rapid energy production confers a competitive advantage because of the limited nutrients to be shared with stromal cells. Moreover, the acidification of the tumor microenvironment due to the secretion of lactate, the final glycolytic product, contributes to cancer cell invasiveness and immune suppression.^174^

However, obtaining energy faster is not the only outcome of the Warburg effect; cancer cells need to maintain a redox state and synthesize building blocks to support tumor proliferation and progression. Thus, in addition to lactate production, some pyruvate is also oxidized into acetyl CoA and enters the TCA cycle, not to obtain ATP but to capture intermediate compounds to use as biosynthetic precursors. For example, citrate is required for fatty acid synthesis with NADPH serving as a cofactor. The loss of intermediates through efflux from the TCA cycle, called cataplerosis, must be resupplied, mainly by glutamine metabolism in a process known as anaplerosis.^176^ Glutamine is one of the most abundant amino acids in cells and is used as the main nitrogen source of cells. Glutaminolysis is upregulated in cancer cells and is required for nonessential amino acid and GSH synthesis, NADPH production, and nucleotide biosynthesis.^175,177^

Furthermore, several glycolysis intermediates are involved in other pathways that are also highly activated in cancer. One of these routes is the pentose phosphate pathway (PPP), which is critical for nucleic acid synthesis, DNA duplication and, consequently, increased biomass production.^175,178^ The first step of the PPP pathway depends on NADP^+^ and is the major source of NADPH in cells. NADPH acts as an essential cofactor with many antioxidant proteins, helping to counteract the oxidative stress produced by the accelerated metabolism, induced hypoxia, and accumulated DNA damage of the cancer cells. Ratios and chemotherapies cause oxidative damage and increase the ROS levels generated. In response to this excessive oxidative stress, some oncogenic factors, such as ATM, Ras, PI3K/Akt/mTOR and Src, upregulate the G6PDH enzyme, activating the PPP pathway.^41,178^ In addition, avoiding the electron transport channel during OXPHOS contributes to the reduction in ROS production in cancer cells.^3^

Another glycolysis-dependent pathway is the serine synthesis pathway, which has been found to be upregulated in cancer and depends on NAD^+^. The glycolytic intermediate 3-phosphoglycerate bypasses glycolysis to produce serine, using NAD^+^ as a redox cofactor. Serine is a precursor of glycine and cysteine and is involved in methionine and folic acid metabolism, amino acid transportation, DNA/histone methylation and redox balance.^179,180^

We can conclude that the main metabolic pathways in cancer, including PPP, serine-based and fatty acid biosynthesis, emerge from the glycolytic pathway. The Warburg effect is the driver of cancer metabolism, for which glucose is the fuel and NAD^+^ is the initiator (Fig. 2).

### NAD^+^ in the EMT and stemness

The pluripotency reprogramming process is closely related to cancer cell dedifferentiation to cancer stem-like cells (CSCs).^72,73^ Tumors are hierarchically organized in different subgroups of cancer cells, which explains their high degree of heterogeneity. At the top of this hierarchy, there is a small subpopulation, called cancer stem cells (CSCs) or tumor/initiating cells (TICs), which are critical not only for tumor initiation and progression but also for therapy resistance and metastasis.^74,181^

NADH has been proposed as a new CSC marker in cancer. Because of its autofluorescence, NADH can be quantified by cytometry,^182^ and interestingly, the NADH^high^ subpopulation has been related to the CD133^+^ CSC subpopulation in glioma.^183^

It has been shown that NAMPT can activate OSKM factors, promoting stemness and dedifferentiation leading to the acquisition of the CSC phenotype in cancer.^7,8,10^ These phenotypes correlate with the transcriptional signature for stemness, such as high ALDH1A1 and aldehyde dehydrogenase activity, the expression of stem-related genes, and CD133 positivity. Mechanistically, NAMPT can also regulate stemness in a paracrine manner through the senescence-associated secretory phenotype.^6–10^ Moreover, through NAMPT activation, SIRT1 is involved in the regulation of several stemness signaling factors, such as the tumor suppressor Hippo pathway,^184^ c-Myc-mediated OCT4 interaction,^78,185^ NICD from the Notch pathway and Gli1 and Gli2 from the Hedgehog pathway.^186^ SIRT1 is also required for genomic stability and telomere elongation during cell reprogramming.^187^

The NAMPT-E2F2-ID1 axis defines the NAD-dependent transcriptional program induced in glioblastoma stem cells. This molecular axis regulates self-renewal and radiotherapy resistance.^188^ NAMPT and NMNAT2 are required for E2F2 expression and self-renewal.

NAD^+^ also regulates epigenetically driven reprogramming. NNMT methyltransferases are essential in methyl group metabolism. NNMT expression is enhanced in mesenchymal stem cells, which promotes an increase in S-adenosyl-methionine, SAM, a methyl donor from methionine. Glioma stem cells show a decrease in SAM and, therefore, in methionine. Glioma stem cells have increased levels of NAD^+^ and hypomethylation, promoting tumors to switch to manifesting mesenchymal properties, increased migration, and a more malignant phenotype.^189^ Downregulating NNMT decreases methyl donor availability and decreases cellular proliferation. This downregulation is followed by a decrease in nonmethylated cytosine levels and an increase in DNMT1 and DNMT3A methyltransferases.^8,64,189^

A comparison of the transcriptomes of the neoplastic and stromal cells in glioma showed 3 different transcriptomic populations.^46^ Among tumor cells, NAMPT was overexpressed in neural tumors compared to neural nontumor cells. This NAMPT increase was also accompanied by CSC markers, such as CD133, CD44, serpin, Jun, or ABCC3. In addition, NAMPT expression was negatively correlated with DNMT1, DNMT3A/3B, and TL3.^8^ These data suggest that an increase in NAMPT and an increase in the levels of NAD^+^ enhanced the malignant phenotype in the cells and led to poorer outcomes.^189^

## NAMPT in cancer

To satisfy high glycolytic and non-glycolitic demands, malignant cells must increase NAD^+^ levels by NAD^+^ biosynthesis upregulation. Since cells rely on the NAD^+^ salvage pathway as the main source of NAD^+^, we have focused on the role of this pathway in cancer.

The rate-limiting step of the salvage pathway is catalyzed by NAMPT and requires PRPP as a cosubstrate, which is mostly supplied by the PPP pathway in cancer cells.^3,190–192^ NAMPT was originally called pre-B-cell colony-enhancing factor (PBEF), a cytokine secreted by early B cells to promote maturation and proliferation. The NAMPT structure presents intracellular and extracellular forms (referred to as iNAMPT and eNAMPT, respectively). eNAMPT correlates with PBEF and visfatin, and it is not surprising to find high eNAMPT levels in different hematopoietic malignancies.^10,193,194^ iNAMPT localizes to the cytoplasm, nucleus and mitochondria, and NAMPT has also been found to be upregulated in several solid human tumors, including the colon cancer,^7^ glioma^8,9^ and breast,^195^ prostate,^196^ thyroid,^197^ and gastric^198^ carcinomas. NAMPT has been described as a potent oncogene able to promote cell proliferation by increasing the NAD^+^ pool, leading to increased tumor progression and development. Many studies have correlated the expression of NAMPT to the clinical outcome of cancer patients. In tumors, such as gastric cancer, glioma and colorectal cancer, higher NAMPT expression correlates with a more aggressive tumor stage, metastasis, treatment resistance, and worse prognosis.^7,8,195,197–200^ In patients with malignant astrocytomas, the level of NAMPT in serum was significantly increased compared to that of the controls.^6–10,201^ In addition, the expression of NAMPT in a molecular environment with mutant p53, IDH mutants or EGFR was associated with the lowest survival of glioblastoma patients.^8,9,46,202^ There are other data suggesting that NAMPT may be a useful biomarker for tumor progression.

However, the proof of concept is based on the analysis of causal effects due to ectopic overexpression of NAMPT in cells. The expression of NAMPT increased tumor cell proliferation, ERK-1/2 and p38 activity levels, the expression levels of metalloproteases, colony formation, and resistance to apoptosis.^8,9,46,203^ Ectopically expressed NAMPT enhanced neovascularization in vivo and enhanced tube formation by human umbilical vein endothelial cells. These effects were mediated by activation of ERK1/2 in endothelial cells, as indicated by ERK inactivation leading to a decrease in these phenomena. The inhibition of PI3K/Akt and ERK1/2 also significantly decreased NAMPT-induced VEGF expression and activation, leading to important inhibition of endothelial cell proliferation and in the formation of new vessels. Ectopic overexpression of NAMPT also activated STAT3. NAMPT promoted the activation of STAT3, upregulating the endothelial IL6 cytokine. The angiogenesis induced by NAMPT was also attenuated after the inhibition of STAT3 activity or the downregulation of the IL-6 function.^204–206^ These activities seemed independent of NAMPT enzymatic activity.

Extracellular eNAMPT inhibited macrophage cell death induced by a number of endoplasmic reticulum (ER) stress activators. eNAMPT rapidly increased IL-6 protein secretion, which activated STAT3-induced prosurvival signals upon autocrine/paracrine binding to the IL-6 receptor.^158^ The ability of eNAMPT to trigger this IL-6/STAT3 antiapoptotic loop was not dependent on the presence of nicotinamide (the NAMPT substrate), was not reproduced by nicotinamide mononucleotide (NMN), the product of NAMPT and was not blocked by the enzyme inhibitor FK866.^158^ A similar effect was observed in glioblastoma and colorectal cancer cell lines, in which the malignant effects of NAMPT overexpression were superior and not reconstituted by saturating amounts of NMN in the cells.^6–10^ Exogenous eNAMPT also enhanced the expression of the adhesion molecules ICAM-1 and VCAM-1 through NF-κB.^205,207,208^ The Induction of NF-κB expression by eNAMPT also induced MMP-2/9 metalloproteinase expression and activation in human vascular endothelial cells.^209–211^ Taken together, these data strongly suggest that the effects of eNAMPT, as a cytokine, are independent of NAMPT enzymatic activity. However, reconstitution of the NAD^+^ pool to some extent and NAD^+^-induced physiological effects have also been observed upon exogenous increases in eNAMPT in glioma and CRC cancer cells.^7^ Although both the increase in the NAD^+^ pool and NAD^+^ effects may happen concurrently, much work is needed to elucidate the role of eNAMPT on NAD^+^.

It has also been reported that NAMPT is able to regulate the canonical WNT pathway.^212^ NAMPT inhibition increases Axin expression, which induces β-catenin degradation. This regulation promotes the inhibition of the WNT signaling pathway, resulting in a decrease in tumorigenesis. The addition of the NAMPT enzymatic product NMN reverses β-catenin degradation. Similarly, the apoptosis induced by the inhibition of NAMPT is partly reversed by knocking down Axin. These results may explain why colorectal cancer tumors show higher levels of NAMPT than are shown by the normal paired samples.

As explained above, the levels of NAMPT are high in many tumor types, and the percentage of tumors with high NAMPT levels with increases in late-stage and metastatic tumors is also high. Furthermore, overexpressed NAMPT levels were associated with poor patient prognoses, independent of tumor stage. The overexpression of NAMPT cDNA in glioma or colorectal cells increased their tumorigenic properties, as well as their acquisition of the CSC phenotype and properties in culture. On the other hand, downregulation of NAMPT mRNA and protein by stable shRNA transfection reduced the tumorigenicity and stemness properties of these cells.^7,8^ NAMPT also activated epithelial-mesenchymal transition pathways, as demonstrated by the activation of several transcription-related factors, such as SNAI1, TWIST, or FOXC2. Increased NAMPT also activated the network of pluripotency transcription factors by activating SOX2, OCT4, KLF4, and Nanog. These findings show that NAMPT overexpression promotes self-renewal properties, resulting in stemness-like maintenance, which ultimately leads to increased migration, dedifferentiation, and CSC-dependent resistance to therapy.^7,8,64^

NAMPT facilitates increases in CSC pools, suggesting that the CSCs in tumors primarily use the NAD salvage pathway to maintain a full, rich source of nutrients for enzymatic activity, leading to tumor progression and the eventual reprogramming of mature tumor cells, which finally results in therapy resistance, relapse, and metastasis. These data also suggest that NAMPT-overexpressing cells have a competitive advantage in nutrient-limiting microenvironments. On the other hand, when the NAMPT product NMN was supplied to cells with low NAMPT expression, the phenotype of the parental cells was restored.^7,8^ NMN may diffuse through the membrane, inducing NMN-deficient production by NAMPT downregulation. In addition, eNAMPT may act biochemically by metabolizing extracellular nicotinamide into NMN, which may diffuse into the cell, restoring the parental phenotype.

NAMPT overexpression induced a transcriptomic signature that was associated with poor survival in human colon cancer or glioma tumors. NAMPT-induced genes associated with effectors of stemness pathways, which increased tumorigenicity and regulated the CSC phenotype. In addition, NAMPT regulation of cancer stem cell pathways correlated with SIRT1 and PARP1, as these proteins seem to play important roles in the CSC phenotype acquisition promoted by NAMPT. These pathway correlations support the hypothesis that NAMPT modulates SIRT1 and PARP1 to control the cellular functions that promote proliferation and stemness pathways, thus regulating the EMT and tumor dedifferentiation. Furthermore, inactivation of these proteins leads to inhibition of both NAMPT signaling and a CSC-like phenotype.^6–10^

Taking all together, NAD^+^ is a central signaling molecule involved in cellular processes implicated in age-related diseases and cancer. NAD^+^ levels are described to decrease during aging, likely through changes in metabolic reactions leading to NAD^+^ synthesis (see before). Models for age-related diseases and show reductions in NAD^+^ pools. NAD^+^ decline seems to be the major feature also in progeria syndromes of accelerated aging, and the administration of NAD^+^ precursors such as NR or NMN offer therapeutic strategies to improve health during aging and progeria comorbidities.^86,125,213,214^ Boosting NAD levels can extend lifespan in organisms and rejuvenate mitochondria which benefits the cardiovascular function, muscle regeneration and glucose metabolism.^87^

However, cancer cells maintain high amounts of NAD^+^ mainly by upregulation of the NAMPT enzyme.^8,64,215^ It is also known that the normal stem cells have large pools of NAD^+^, similar to cancer cells,^188^ and the upregulation of NAMPT in these tumor cells with concomitant raise of NAD^+^ levels de-differentiate these cancer cells to CSC like cells.^8,64,215^ It is possible that, with aging, the decrease in NAD^+^ not only affect the mature tissue but also stem cells, losing regenerative capability. An increase in NAD^+^ levels in normal/stem cells could recover part of their capabilities allowing improve in general health. However, the recovery of these regenerative capabilities (by increased expression of NAMPT for example) change the physiology of the cells towards stem-like cells, gaining some cancer properties, such as unlimited proliferative capacity, altered metabolism, DNA-repair, methylation alterations, with phenotype similar to CSCs, which deregulation by any other mutation initiate the tumor.

## NAPRT in tumors

There is a controversy regarding the expression levels of NAPRT, the limiting enzyme of The Preiss–Handler pathway, in cancer. In ovarian,^216^ prostate and pancreatic cancers, NAPRT has been found upregulated just like NAMPT gene.^193,217^ While in glioblastoma, neuroblastomas,^218^ chondrosarcoma,^219^ gastric cancer,^220^ leukemia^193^, and colorectal cancer,^221^ NAPRT has been found downregulated.

Comparing the expression pattern of NAPRT and NAMPT gene in human tumor cell lines versus normal tissues, the researchers found a great variability in mRNA and protein levels of NAPRT in tumor cells.^222^ This variability depends on NAPRT expression level of the original tissue. Tumors arising from normal tissues that highly express NAPRT will have high frequency of NAPRT amplifications, while tumors arising from low NAPRT expression tissues will depend completely on NAMPT activity.^217,221,223^

Moreover, NAPRT expression can be modulated by transcriptional and post-transcriptional mechanisms more frequent than NAMPT. Several alternative NAPRT transcripts have been detected in some tumors. These transcripts are tissue-specific, have different functional consequences but no recognizable polyadenylation signal in their 3’UTRs, and they are tissue-specific.^221,222^ NAPRT splicing alternative can be regulated by several RNA binding proteins (RBPs), including Argonaute and neuro-oncologic ventral antigen 1 (NOVA-1). It has been detected six binding sites for Argonaute proteins, which are transcriptional silencers and are members of RNA-induced silencing complex (RISC), a key regulator of splicing alternative; and two binding sites for NOVA-1, which is a specific RBPs protein in brain and can recognize YCAY motifs of pre-mRNA.^222,224^

In addition, it has been shown that NAPRT gene promoter can be hypermethylated resulting in the inhibition of NAPRT epigenetic expression in some types of tumors.^221^ This is the case of MKN28 cancer cell line, where no mRNA nor protein expression was found due to high methylation of NAPRT promoter region.^222^ Higher NAPRT promoter methylation has been significantly correlated with higher grade of tumor malignancy in chondrosarcoma.^219^

NAPRT negative tumors have in common that they present mutations in isocitrate dehydrogenase 1/2 (IDH). IDH1/2 mutants use NADPH to transform α-ketoglutarate (α-KG) into D-2-hydroxyglutarate (D-2HG) which can act as an oncometabolite. It has been shown that D-2HG suppress NAPRT expression by hypermethylation at CpG island of the NAPRT promoter.^219,225,226^ Recently, it has been reported that the Protein Phosphatase Mg^2+^/Mn^2+^ Dependent 1D (PPM1D) can also inhibit NAPRT expression. PPM1D is an oncogene involved in DNA damage response and cellular checkpoint pathways. Mutations in PPM1D gene results in hypermethylation of CpG islands of NAPRT promoter, thus suppressing NAPRT expression.^227^

NAPRT activity inhibition and, consequently, the blockade of The Preiss Handler pathway force cancer cells to completely depend on the salvage pathway. Therefore, it is not surprising that NAMPT is upregulated in these tumors, since cancer cells depend on its activity to satisfy their high NAD^+^ demand.^226^ Interestingly, IDH1/2 and PPM1D mutants seem to be sensitive to NAMPT inhibitors treatment. The loss of NAPRT activity makes cancer cells more vulnerable to the effect of NAMPRT inhibitors due to the total blockage of NAD^+^ production. Thus, NAPRT negative tumors are highly dependent on NAMPT activity, making them perfect candidates for treatment with NAMPT inhibitors.^225–227^ Low levels of NAPRT expression has been reported in most EMT-subtype gastric cancer, which is positive for EMT markers and is associated with poor prognosis and survival in patients. Since NAPRT is required to EMT inhibition through β-catenin, NAPRT suppression can promote EMT in gastric tumors.^220^

However, the total depletion of NAD^+^ could be very toxic to noncancerous cells, thereby it has been considered to rescue them using NAPRT precursor, nicotinic acid. Nicotinic acid treatment may help activate NAPRT, thus protecting noncancerous cells from the drastic NAD^+^ depletion by NAMPT inhibitors.^193,217^ Several NAMPT inhibitors have been tested in combination with nicotinic acid. For example, the coadministration of nicotinic acid helps to prevent retinal and hematological toxicities of LSN3154567.^228^ GMX1777 and nicotinic acid co-treatment results in an increase of the antitumoral affects in xenograft tumors derived from NAPRT negative cell lines.^218^ However, the inhibition effect of STF-31 was partially suppressed by nicotinic acid in NAPRT positive cell lines.^229^ Indeed, it has been shown that nicotinic acid can rescue the inhibitory activity of NAMPT inhibitors in those cancer models that express NAPRT.^228^ Therefore, the co-administration of nicotinic acid and NAMPT inhibitors has been proposed as a new therapeutic strategy but only in NAPRT negative cancers.^193,217^

On the other hand, NAPRT has found overexpressed in some tumors where it has been involved in cell metabolism and DNA repair. For example, in colorectal cancer, NAPRT and NAMPT have been proposed as negative prognostic value, since high levels of both genes are associated with invasion, metastasis, and worst prognostic.^221^ In ovarian cancer, high NAPRT expression levels correlate with the deficit of BRCA-2.^216^

In NAPRT positive tumors, cancer cells have an adequate supply of NAD^+^, allowing PARP activation which is required for oxidative and DNA damage protection.^216,230^ Thereby, NAPRT overexpression can contribute to resistance to NAMPT inhibitors and DNA-damaging drugs treatment. In vivo, it has been observed that xenografts tumors are more resistant to FK866 treatment when NAPRT is upregulated, while they became more chemosensitive when NAPRT is silenced or inhibited. Several compounds have been identified as NAPRT inhibitors, e.g., 2-hydroxinicotinic acid (2-HNA)^216^ which has been shown to be able to enhance the inhibitory effect of FK866.^221^ Depending on the type of cancer to be treated, NAPRT may be a predictive biomarker of the NAMPT inhibitors treatment success.

## SIRT in cancer

**Sirtuins** (SIRT1–7) are a family of deacetylase proteins for which NAD^+^ is a coenzyme for the removal acetyl modifications of lysine residues on histones and other proteins, generating o-actyl-ADP-ribose and NAM as the reaction products.^59,70,105^ The best characterized member is SIRT1, which has been associated with longevity. There is evidence showing that resveratrol, a potent antioxidant with anti-aging effects, is able to increase NAD^+^ levels, leading to SIRT1 activation.^59,70,231^

The NAD^+^-consuming enzyme SIRT1 strongly depends on the NAD^+^/NADH ratio and can be inhibited by high NAM levels and DBC1, an endogenous SIRT1 inhibitor. Most of the evidence shows that SIRT1 can act as a tumor promoter; however, there is some evidence that suggests it is a tumor suppressor. It is well known that SIRT1 has an anti-aging effect and that its activity can be increased by calorific restriction, which can also reduce cancer risk.^1,41^ SIRT1 also acts as a deacetylase and inhibits HIF-1α, one of the factors needed to activate the Warburg effect.^174^

However, SIRT1 has been found to be upregulated in several human cancers. The relationship between SIRT1 and NAMPT has been well established; that is, NAMPT promotes SIRT1 activity through a NAD^+^ pool increase and NAM level reduction.^232^

In addition to HIF-1α, SIRT1 regulates other important factors, such as p53, c-Myc, FOXO3, E2F1, BAX, and NF-κB. The most important antitumoral effects of p53 are cell cycle arrest and apoptosis. P53 can also inhibit G6PDH in the PPP pathway. SIRT1 deacetylates and inhibits p53, ensuring R5P and NADPH production, and promotes cell survival.^5,233^ c-Myc is a proto-oncogene that can be activated through the PI3K/Akt/mTOR pathway. C-Myc regulates many genes involved in several metabolic pathways, such as glycolysis, lactate production, glutaminolysis, and fatty acid synthase.^175^ C-Myc also regulates SIRT1 activity by inducing NAMPT expression and inhibiting BDC1. SIRT1 activates c-Myc by deacetylation via positive feedback.^232^ Moreover, SIRT1 deacetylates and activates FOXO3, which contributes to oxidative stress resistance by upregulating antioxidant proteins.^196,234,235^ SIRT1 is also involved in the promotion of cancer cell proliferation, angiogenesis, and metastasis through the activation of the MAP kinase pathway by deacetylation.^234,235^ All these data corroborate the oncogenic role of SIRT1, which strongly depends on the NAD^+^ pool and NAMPT activity.

Another major cytosolic NAD^+^-consuming enzyme is SIRT2, originally considered a tumor suppressor.^236^ SIRT2 inhibits the peroxidase activity of peroxiredoxin-1 (Prdx-1) by deacetylation, increasing oxidation, and sensitizing breast tumor cells to further increase ROS.^237^ Another target of SIRT2 is HIF1α in the cytosol, promoting the hydroxylation and degradation of HIF1α and suppressing the tumor growth dependent on hypoxia.^238^ However, other works have demonstrated that SIRT2 promotes cancer growth by stabilizing the Myc family of oncoproteins^239,240^ and enhancing the signaling of the Notch pathway.^241^ Mutations in the *SIRT2* gene have also been reported in human cancers, impairing genome maintenance and promoting tumorigenesis.^242,243^ SIRT2 has also been described as a sensor for the amount of nutrients available in the cell and regulating metabolic activity to adapt the energy need for cancer growth.^244^

SIRT3, similar to SIRT4, has both oncogenic and tumor-suppressive activities.^245^ SIRT3 is localized in the mitochondria but is also localized in the nucleus and translocated to mitochondria upon DNA damage. This translocation contributes to the de-repression of genes involved in mitochondrial function.^246^ SIRT3, on the other hand, inhibits apoptosis and promotes cell growth, increases glycolytic metabolism,^247^ maintains mitochondrial membrane integrity,^248^ promotes mitochondrial DNA repair^249–251^ and increases cell resistance to environmental stress.^252–254^ SIRT3 can act as a tumor suppressor by inactivating HIF1α and decreasing oxidative stress.^255,256^ It can also activate FOXO3A and therefore suppress the EMT in prostate cancer cells.^257^ SIRT3 activation correlates with an increase in mitochondrial NAD^+^ and a number reduced of glioblastoma-initiating cells.^258^ In contrast, the reduction of mitochondrial NAD^+^ levels inhibits SIRT3 activity, increasing ROS levels through the deactivation of superoxide dismutase 2 (SOD2), facilitating the metastasis of hepatocellular carcinoma cells.^259^ SIRT4 functions as an ADP-ribosyltransferase, promoting tumorigenicity when induced under oncogenic stress^260^ and maintaining metabolic homeostasis.^261^ On the other hand, SIRT4 suppresses tumor growth by inhibiting glutamine metabolism,^262,263^ and upon downregulation, it inhibits apoptosis and desensitizes cancer cells to chemotherapy.^264^

SIRT5 and SIRT6 are master regulators involved in metabolic reprogramming during tumorigenesis.^265^ SIRT5 regulates the NAD^+^-dependent elimination of the succinyl, glutaryl, or malonyl groups from lysine residues and, in contrast, is an inefficient deacetylase.^266^ SIRT5 confers chemotherapy resistance to CCR cells through the elimination of malonyl groups from succinate dehydrogenase complex subunit A (SDHA). SIRT6 also suppresses the Warburg effect through the inhibition of the pyruvate kinase M2 (PKM2), thereby regulating glucose homeostasis.^267,268^ SIRT6, on the other hand, maintains genome integrity during tumorigenesis.^269^ In this regard, high SIRT6 levels have been reported in colon cancer samples, which correlate with worse prognosis.^270^

The role of SIRT7 in cancer is poorly understood. SIRT7 regulates RNA metabolism and the biogenesis of ribosomes. SIRT7 deacetylates CDK9, a subunit of the P-TEFb elongation factor,^271,272^ leading to the activation of RNA polymerase II-dependent transcription. SIRT7 is also a prognostic factor indicating a poor outcome in colorectal cancer because it induces the EMT and cell invasion.^273^ However, SIRT7 inhibits breast cancer metastasis by promoting SMAD4 degradation and antagonizing TGF-β signaling.^274^

## NAMPT-NAD^+^ and AMPK

In response to a metabolic stress state, e.g. caloric restriction, low glucose availability, exercise, and ischemia, cells can promote sirtuins activation by increasing NAD^+^-production as previously described. But also, other important metabolic regulators, such as AMP-activated kinase (AMPK), can be activated to counteract energy deprivation.^275^

An enhanced AMP/ATP rate induces the activation of AMPK which can regulate different metabolic enzymes, transcriptional factors and histones acetylation by phosphorylation. This signaling pathway switches on catabolic processes promoting ATP production and turns off anabolic processes avoiding energy consumption.^275,276^ Therefore, AMPK can activate glycolysis, fatty acid oxidation and, also, NAD^+^ metabolism.^277^ Indeed, AMPK has been involved in the modulation of NAD^+^/NADH ratio affecting the activity of SIRT1, a NAD^+^-consumed enzyme, which is another important metabolic sensor in cells.^276^ AMPK can modulate SIRT1 activity in a direct and indirect way. Thus, SIRT1 is a direct target and it can be directly phosphorylated by AMPK. On the other hand, AMPK can phosphorylate GAPDH which, once phosphorylated, is translocated to the nucleus where is able to inhibit DBC1, an SIRT1 endogenous inhibitor, increasing activated SIRT1 levels.^275,278^

In addition, AMPK can promote SIRT1 activity via NAMPT activation. Since NAMPT, in the rate-limiting reaction of NAD^+^ salvage pathway, consumes NAM, which acts as an endogenous inhibitor of SIRT1, to regenerate and boost NAD^+^ pool, thus promoting SIRT1 activation.^276,277^ It has been shown that AMPK activation is required to induce NAMPT transcription during glucose deprivation in skeletal muscle cells. AMPK may phosphorylate and activate FOXO transcription factors that induce NAMPT expression.^275,279^ The results of others studies support this hypothesis, since high NAMPT protein and mRNA levels were observed when muscle cells were treated with the AMPK activator AICAR (5-Aminoimidazole-4-carboxamide ribonucleotide).^277,280^

Liver kinase B1 (LKB1) is an upstream kinase of AMPK and, also, is a target of SIRT1. Thus, SIRT1 can deacetylate LKB1 resulting in AMPK activation, closing a positive feedback loop between AMPK and SIRT1.^275,281^ CD38 also seem to be involved in AMPK activation, as it is the major NADase in cells.^27^

Cellular metabolism is completely reprogrammed during tumorigenesis, resulting in deregulation of AMPK signaling. LKB1, which activates AMPK, has been described as tumor suppressor.^282,283^ AMPK acts as checkpoint in the regulation of mTOR, p53, and other signaling molecules involved in cell growth, polarity, survival, autophagy, and apoptosis. It is not surprising to find AMPK downregulated in many human malignancies, whereas NAMPT and mTOR are found upregulated.^282,284,285^ Some AMPK activator, such as AICAR and metformin, have been shown to inhibit tumor proliferation in melanoma and hepatocarcinoma.^285,286^

NAMPT inhibition in cancer has been associated with an increase of AMPK activity. Since NAD^+^ is required for ATP production, FK866-induced NAMPT inhibition affects ATP production and, consequently, results in AMPK activation (in hepatocarcinoma cells).^286,287^ This AMPK activating effect has been favored in IDH1 mutant cells.^225^ According to these studies, NAMPT inhibitors and AMPK activators combination could be an interesting option for use in therapy.

## PARPs in cancer

**PARPs** or poly(ADP-ribose) polymerases are DNA repair nuclear proteins that can transfer ADP-ribose from the coenzyme NAD^+^ to protein substrates. The reaction products are poly-ADP-ribose (PAR) chains and NAM.^70^ PARP1 is the best characterized and most studied member that is involved in repairing single strand breaks (SSBs). PARP1 is also known as the guardian of DNA integrity since it is activated in response to DNA damage. However, excessive PARP1 activation, due to exposure to genotoxic agents, can dramatically reduce NAD^+^ and ATP levels, affecting sirtuin activity and cellular homeostasis and leading to cell death.^5,70^

The main function of PARP1, another NAD^+^-consumer enzyme, is DNA repair, but it is also involved in cell proliferation, cell cycle regulation, gene transcription, inflammation, and cell fate. DNA damage and high levels of NAD^+^ can activate PARP1 in cells. Depending on the degree of its activation, PARP1 can act as a tumor promoter or suppressor. In the case of severe DNA damage, PARP1 drives apoptosis through p53 activation under normal conditions.^5,288^

However, most tumors upregulate PARP1 to support DNA damage, which is enhanced by radio- and chemotherapies. Indeed, higher PARP1 expression has been found in several carcinomas, including breast, ovarian, and lung cancers and non-Hodgkin’s lymphoma.^10,288^ PARP1 can also stimulate ERK activity, promoting proangiogenic and metastatic factors. Some PARP inhibitors, such as olaparib, have been studied and tested in clinical trials since cancer cells seem to be more sensitive than noncancerous cells. A deficit of alternative DNA repair mechanisms in cancer cells may explain this enhanced sensitivity. However, tumor cells can develop resistance to PARP1 inhibition, and further studies are needed to understand this mechanism.^41^

PARP1 may have both oncogenic and tumor suppressor functions in colorectal cancers. It has been reported ^289^ that PARP1 suppresses tumor initiation after DNA alkylation but also promotes tumor progression driven by inflammation. PARP1 may regulate the E2F1 transcription factor by poly-ADP-ribosylation, reducing the apoptosis rate of cancer cells that had been induced by E2F1 activation.^290^ Activated PARP1 also possesses a transcription-regulatory function, promoting the induction of DNA repair factors by E2F1 and enhancing the progression to treatment-resistant cancer.^291–293^ For an in-depth discussion of the localization and function of other PARP enzymes, we refer readers to these works.^294,295^

## cADPRSs in cancer

**cADPRSs** or cyclic ADP-ribose synthases constitute a nuclear protein family of glycohydrolases, including CD38 and its homologs CD157, CD39, and CD73. These proteins cleave NAD^+^ into NAM, ADPR, or cyclic ADP-ribose (cADPR). The latter is a secondary messenger involved in calcium mobilization, cell cycle control, and insulin signaling.^4,70^ CD38 is the major NAD^+^ consumer in mammals; indeed, 100 molecules of NAD^+^ are needed to generate 1 molecule of cADPR.^5,296^ Recently, a newly discovered enzyme with NAD^+^-hydrolyzing activity called SARM1, which also generates cADPR and NAM, has been reported.^297^

The most studied cADPRS is CD38, which is the major DNase in cells. CD38 has been described as a negative prognostic marker in chronic lymphocytic leukemia and as a surface marker in multiple myeloma^41,298^ In addition, CD38 may regulate the sensitivity of pancreatic cancer cells to FK866 treatment.^234,299^ However, the relationship among NAMPT, NAD^+^, and CD38 is still poorly understood, and more studies are needed. CD38 and its homolog CD157 are being studied as possible cancer targets, and antibodies and inhibitors are being developed.^26^

The aging-related NAD^+^ decrease is partly due to an increase in CD38 expression.^123^ CD38 downregulates NAD^+^ by converting NAD^+^ to cyclic-ADP riboside, mobilizing intracellular or extracellular Ca+. Interestingly, the carboxyl-terminal catalytic domain of CD38 media has two opposing orientations and is therefore able to mobilize either intra- or extracellular NAD^+^ pools.^300^ However, CD38 expression specifically leads to the consumption of extracellular NAD^+^ and not intracellular NAD^+^ in prostate cancer cells.^23^ Moreover, CD38 also consumes extracellular NMN, generating NAM, which can be converted to NAD^+^ through NAMPT and NMNAT after crossing the plasma membrane.^123^ Another important membrane-bound enzyme that degrades extracellular NAD^+^ is CD73. CD73 successively degrades NAD^+^ to NMN and further to NR.^301,302^ The conversion of extracellular NMN to NR is important to support intracellular NAD^+^ biosynthesis.^302,303^ CD73 also functions as an adhesion molecule that mediates cancer cell migration and invasion.^304^

## NAMPT use as a therapeutic strategy

The inhibition of NAMPT may lead to the depletion of NAD^+^, which in turn inhibits ATP synthesis. This effect eventually causes the attenuation of cancer cell proliferation and death. Therefore, NAMPT has been proposed as a promising therapeutic target. As potent anticancer targets, several specific inhibitors have been developed, and currently, some of them are in phase II clinical trials.^3,10,194,234^ The most studied inhibitor is FK866/APO866, which targets NAMPT in a noncompetitive manner and has shown significant anticancer effects. NAMPT inhibition by FK866 is important, leading to NAD^+^ depletion, which causes a reduction in glucose uptake, ATP production, fatty acid synthesis, an increase in ROS levels and affects the activity of all NAD^+^-dependent enzymes, such as sirtuins and PARPs. All these effects lead to blocked proliferation and cell death through apoptosis.^234^ In a phase I study in advanced solid tumors, thrombocytopenia was found to be the dose-limiting toxicity. However, no objective tumor responses were observed.

FK866 treatment does not affect the NAD^+^ mitochondrial pool; therefore, cancer cells may be more sensitive than noncancerous cells to FK866.^194^ Moreover, FK866 cytotoxicity seems to be higher in tumors that express higher NAMPT levels and are relatively more glycolysis-dependent.^223,234^ NAMPT is also essential for normal cells; therefore, the adverse side effects of NAMPT inhibitors are important in clinical trials. Recent advances have attempted to target NAMPT at suboptimal doses in combination with other compounds and developing good expected activity.^7,8,64^

FK866 was tested in mass cultures and 3D tumorspheres enriched with cancer stem cells from human colorectal cell lines. NAMPT-overexpressing cells, either in the mass culture or tumorspheres, were more sensitive to FK866 than were parental cells or cells with reduced levels of NAMPT.^7^ (FK866 was effective against this NAMPT-dependent cancer stem cell pool, as indicated by the reduced dose of the drug necessary to induce cell death. Interestingly, this treatment combined with either sirtinol or olaparib enhanced the sensitivity of the cells against FK866 both in mass cultures and tumorspheres, suggesting that these combinations can be used to reduce the toxic effects of NAMPT inhibition during the incorporation of a more effective therapy.^7^ Thus, NAMPT inhibitors, in combination with sirtinol or PARP inhibitors, may represent a new therapy for patients with colon cancer.

In addition, in colorectal cancer and glioblastoma, stable NAMPT-overexpressing cells were substantially more resistant to the apoptosis induced by diverse chemotherapeutic treatments than were their parental counterparts.^6–10^ In contrast, the cells with NAMPT knocked down, downregulating NAMPT expression, were more sensitive to therapy than were the controls.^6–10^ NAMPT inhibitors in clinical trials also reduced the growth of cisplatin-treated xenograft tumors in ovarian cells in vivo. Moreover, a combination of NAMPT inhibition and cisplatin improved the survival of mice with ovarian tumors.^305^ In addition, therapy-induced cellular senescence increases resistance to therapy by inducing a CSC-like phenotype. NAMPT inhibition suppresses the senescence-associated CSC increase and resistance induced by cisplatin in ovarian tumors.^305^

Similarly, NAMPT-overexpressing cells were more sensitive to FK866 in mass cultures than were parental cells or cells with downregulated NAMPT. FK866-induced toxicity was higher in the CSC-enriched cultures because of the high NAMPT level. Interestingly, the combined treatment with TMZ slightly increased the sensitivity values of this CIC population, suggesting that this combination is an effective therapy.^8,225,306^ Thus, NAMPT inhibitors may sensitize cells to standard therapies in glioma CIC populations, particularly in patients expressing high levels of the gene signature. In glioblastoma, specific genetic and transcriptional subgroups might be differentially sensitive to the inhibition of NAMPT.^7,8,64,225,306,307^ IDH mutations sensitize CSCs of glioblastoma to NAMPT inhibition due to low levels of NAPRT1, resulting in low NAD levels. Myc or NMyc increase gene expression in glioblastoma, including those involved in glycolytic flux, and reduces the NAD^+^ pool, leading to increased glioma cell sensitivity to NAMP inhibition.^225,306^

FK866 treatment does not affect the NAD^+^ mitochondrial pool; therefore, cancer cells may be more sensitive to FK866 than noncancerous cells.^194^ Moreover, FK866 cytotoxicity seems to be higher in tumors that express higher NAMPT levels and in cells that depend more on glycolysis.^46^

Recently, KPT-9274 was discovered as a novel orally available NAMPT inhibitor. This compound is currently in phase 1 clinical evaluation in patients with advanced solid malignancies or non-Hodgkin’s lymphoma (NHL) (clinicaltrials.gov; NCT02702492)^308–310^ (Table 1). KPT-9274 was originally found as a small molecule that binds and destabilizes PAK4 protein in cells.^308,311^ KPT-9274 was found to be also capable of inhibiting NAMPT enzyme.^311^ Thenceforth, this inhibitor is been studied in human tumors which are NAMPT and PAK4 upregulated. It has been shown that KPT-9274 can promote apoptosis and induce DNA damage in vitro,^312^ and significantly reduces tumor proliferation and survival in xenograft models in different tumors, such as pancreatic cancer,^309^ pancreatic neuroendocrine tumors,^313^ triple-negative breast cancer^311^, and leukemia.^314,315^

**Table 1:** NAMPT inhibitors in clinical trials

NAMPT inhibitor	Clinical trials. Gov identifier	STUDY START DATE	Description	Results
**KPT-9274/ ATG-019**	NCT04281420	2020 (Recruiting)	**Phase 1**: Open-Label Study of the Safety and Tolerability of ATG-019/KPT-9274, a Dual Inhibitor of PAK4 and NAMPT, in Patients with Advanced Solid Tumors or Non-Hodgkin’s Lymphoma.	–
	NCT02702492	2016 (Recruiting)	**Phase 1**: Open-Label Study of the Safety, Tolerability, and Efficacy of KPT-9274 a Dual Inhibitor of PAK4 and NAMPT, in Patients with Advanced Solid Malignancies or Non-Hodgkin’s Lymphoma.	–
**OT-82**	NCT03921879	2019 (Recruiting)	**Phase 1**: Safety and Efficacy of OT-82 in Participants with Relapsed or Refractory Lymphoma.	–
**APO866/ FK866**	NCT00435084	2007 (Completed)	**Phase 1/2**: Study to Assess the Safety and Tolerability of APO866 for the Treatment of Refractory B-CLL.	Treatment was safe and well tolerated. DLT was thrombocytopenia. A decrease of serum VEGF levels was observed 96 h after the start of treatment at MTD.
	NCT00431912	2007 (Completed)	**Phase 2**: A Study of APO866 for the Treatment of Cutaneous T-cell Lymphoma.	No objective tumor response. However, 4 patients of 24 had stable disease. 80% size reduction of melanoma was observed.
	NCT00432107	2006 (Completed)	**Phase 2**: A Study to Assess APO866 for the Treatment of Advanced Melanoma.	
**CHS-828/ GMX1778**	NCT00003979	1999 (withdrawn)	**Phase 1**: CHS 828 in Treating Patients with Solid Tumors.	Critical toxicity. Thrombocytopenia and gastrointestinal symptoms^338^

*DLT* dose-limiting toxicity, *MTD* maximum tolerated dose. https://clinicaltrials.gov/ct2/home

KPT-9274 may target to cancer stem cell population, since NAMPT and PAK4 proteins play an important role in EMT activation.^309^ Interestingly, in B-cell acute lymphoblastic leukemia (B-ALL) and acute myeloid leukemia (AML) models, the effect of KPT-9274 can be reversed by treating the cells with NAD^+^ precursors, for example nicotinic acid. These results suggest that the inhibitory effect seem to be more dependent on NAD^+^ depletion than on PAK4 blocking.^314,315^ The dual inhibition of KPT-9274 allows targeting of NAD^+^ regeneration and PAK4 signaling, which are essential for tumor development and survival, making KPT-9274 an attractive drug candidate for use in chemotherapy.^308,316^

OT-82 is another novel NAMPT inhibitor and has been shown to be more efficiency in hematological malignancies than solid tumors 20. OT-82 promoted the efficacy of other drugs such as cytarabine and dasatinib. OT-82 resistance was related to a high expression of CD38 in ALL cells.^317,318^ In preclinical studies, OT-82 was well tolerated and showed no cardiac, neurological, or retinal toxicities in toxicological studies in mice and nonhuman primates.^318^ Currently, OT-82 is in phase 1 clinical evaluation of safety and efficacy in participants with relapsed or refractory lymphoma (clinicaltrials.gov; NCT03921879, Table 1)

Other NAMPT inhibitors, including LSN3154567, STF-31, STF-118804, GNE-617, and CB 300919, have not entered clinical trials due to their high toxicity or low safety.

Other small molecule-specific inhibitors of NAMPT, such as GMX1777 and GMX1778, showed anticancer effects similar to the effect of FK866. GMX1777 is a cyanoguanidine compound that is rapidly converted into the active form GMX1778, a competitive inhibitor of NAMPT. The combination of radiotherapy and GMX1777 has shown therapeutic efficacy in a mouse model of HNCC. In a phase I study of advanced resistant solid tumors, GMX-1778 (CHS-828) induced gastrointestinal toxicity and thrombocytopenia. No objective tumor responses were observed. GMX-1777 is also in a phase I study of advanced malignancies. Thrombocytopenia and gastrointestinal hemorrhage limited the dose. No objective tumor responses were observed.

STF-31 is a small-molecule inhibitor of NAMPT that selectively kills renal cell carcinoma cells upon the loss of the von Hippel-Lindau (VHL) tumor suppressor gene. STF-31 inhibits glucose uptake and the Warburg effect, and NAMPT-H191R mutations confer resistance to STF-31 treatment. STF-31 in cancer cell lines also targets Glut1, and it has been proposed that STF-31 also exerts an additional inhibitory effect because dual functions are beneficial for inducing cell death in tumors with a high glucose consumption rate. The small-molecule LSN3154567 is an orally available compound that, when coadministered with the NAD^+^NAD^+^ precursor nicotinic acid, does not lead to the hematological toxicity found in with the use of other NAMPT inhibitors.

Cancer cells can develop resistance to NAMPT inhibitors in many ways. *NAMPT* mutations^319^ enhance NAD^+^ synthesis through other pathways, such as the *de novo pathway*, upregulate quinolinic acid phosphoribosyltransferase (QAPRT), and enhance amino acid catabolism (for example, tryptophan in the *de novo* pathway or glutamine in the salvage pathway).^320^ Moreover, resistance to cancer therapy has been attributed to the self-renewal and dedifferentiation of cancer stem cells (CSCs) residing within the tumor.^321^ CD133-high/CD44-high populations of glioma cell lines are enriched in NAMPT, correlating with glioma stem cells. NAMPT expression also correlates with high Nanog levels in patient-derived glioblastoma tumors,^8^ pointing to the role of NAMPT in maintaining the treatment-refractory component of glioblastoma. Metabolic and oxidative stress have been suggested to be the cause of the resistance of CSCs to therapeutic treatment because of the metabolic plasticity induced in CSCs; however, these CSCs have also been reported to be highly sensitive to compounds that target mitochondrial metabolism.^76,322–324^ Therefore, mitochondrial targeting of NAMPT activity may be used to avoid the toxicity observed in first-generation inhibitors and provide an enhanced therapeutic window. Cancer cells undergoing stress (for example, oxidative stress, glucose deprivation or hypoxia) generate the mitochondrial signaling molecule hydrogen sulfide (H_2_S), which has the ability to upregulate NAMPT.^325,326^ The dedifferentiation induced by H2S in cancer cells can be suppressed by NAMPT inhibition. Furthermore, H2S-producing enzymes can also be upregulated by NAMPT.^325^ The feedback between NAMPT and other signaling pathways also provides tumor-specific opportunities for combinatorial therapeutic targeting of NAMPT. Along this line, because of synergistic effects between NAMPT and HDAC inhibitors, dual NAMPT/HDAC inhibitors have been developed and have shown good efficacy in vitro. These dual compounds induce apoptosis and autophagy in cancer cell lines and cytotoxicity in xenograft tumor models in vivo.^327,328^ Combining FK866 with the PARP inhibitor, olaparib, results in cell death through the suppression of the NAD^+^ serving as the substrate of PARP in triple-negative breast cancer or colon cancer cell lines as well as in vivo models, with the combinatorial treatment inducing negligible systemic.^46^ Thus, the true utility of NAMPT as a cancer target may be in synergistic combinations that enable more effective targeting of known cancer-driving pathways by regulating the NAD^+^-dependent biology in these pathways. The added advantage of these combinatorial approaches is that their tumor specificity may abrogate the systemic toxicity associated with single-agent by reducing dose requirements or may enable better therapeutic index in tumors compared to normal tissue. Interestingly, an underdevelopment in this field: exploration of NAMPT inhibitors in combination with inhibitors of immune checkpoints.

### NAD in immunotherapy

The macrophage colony-stimulating factor MCSF increased NAMPT levels in myeloid cells, mobilizing myeloid-derived suppressor cells and contributing to immunosuppression in the tumor site. In addition, NAMPT inhibition decreased the number of MDSCs and resensitized tumors to immunotherapy in mouse models in preclinical studies.^154^

NAMPT is also increased in tumor-associated neutrophils (TANs). The inhibition of NAMPT ex vivo in TANs and the transfer of these cells to mice reduced tumorigenesis through the suppression of Sirt1 inhibiting proangiogenesis.^329^ The evidence linking myeloid cell fate and the NAD-NAMPT axis suggests an important mechanism for reprogramming myeloid cells through NAMPT (either extracellular or intracellular) inhibition. In the context of cancer, an immunosuppressive microenvironment is generated when NAMPT inhibitors are given in combination with immunotherapy.^138,330^

Intracellular NAMPT, activated through sirtuins, regulates the metabolic reprogramming of myeloid cells and tumor cells under hypoxia. However, extracellular NAMPT induced through the TLR4 receptor activates signaling that triggers the differentiation and secretion of anti-inflammatory and tumorigenic cytokines and creating an immunosuppressive microenvironment. The inhibition of both NAMPT activities can repolarize myeloid cells and inhibit tumor growth, especially in combination with immune checkpoint therapies. These and other results suggest that the inhibition of NAMPT can be used to increase the efficacy of immunotherapies.

Tregs with high expression of CD38 have been observed in multiple myeloma and in ALL. In these tumors, the elimination of cancer cells with high CD38 levels is associated with an increase in helper T and cytotoxic T lymphocytes and a functional T-cell response.^331,332^ In these tumors, the use of monoclonal antibodies against CD38 is a promising therapy for the recovery of immune surveillance. A functional relationship between the subset of Th17 immunosuppressive TILs and Cd38+ expressing cells has also been described,^32^ and the targeting of subpopulations with high CD38 expression can also restore tumor control via adoptive cell therapy (ACT). or administering immunomodulatory drugs. CD38 was also considered a major contributor to the acquired resistance to PD1/PDL1 blockade, causing CD8+ T-cell suppression.^46^ The combined targeting of both CD38 and PDL1 enhances the antitumor immune response. CD38 inhibition regulates CD8+ T-cell proliferation and the secretion of cytokines with antitumor properties and restores CD8+ T-cell killing capability.^333^

Tryptophan catabolism was found to contribute to the escape of the immune response through accumulated catabolites, creating an immunosuppressive microenvironment in tumors and lymph nodes, with the catabolites binding to and activating AhR.^334^ This process leads to the induction of T-cell anergy, cell death, the differentiation of CD4+ cells into Tregs and polarization of macrophages to acquire an immunosuppressive phenotype.^46,335^ High levels of IDO, the enzyme involved in the catabolism of tryptophan, have been detected in many types of tumors associated with poor response. According to its role in immunosuppression, IDO has also been proposed as a target for cancer therapy.^336,337^ The inhibitors of IDO are currently under investigation in clinical trials for solid cancer to enhance primary anticancer therapy, including antibodies and immune checkpoint inhibitors. The IDO inhibitors are in development for use in overcoming the resistance to immunotherapies targeting immune checkpoints, thus supporting combination therapies.

## Conclusion and perspectives

NAD^+^ is an essential metabolite for cellular homeostasis. Multiple redox metabolic reactions depend on it to obtain energy. Moreover, NAD^+^ acts as a substrate of sirtuins, PARPs, and cADPRSs in many different signaling pathways, including DNA repair, posttranslational modifications, inflammatory responses, senescence, and apoptosis.

NAD^+^ depletion leads to degenerative diseases, including aging, while an increase in the NAD^+^ pool has been related to tumorigenesis. Cancer cells shift from normal OXPHOS to glycolysis metabolism to obtain a faster ATP production rate, maintain redox balance and synthesize nucleic acids, proteins and fatty acids. This reprogramming metabolism, also called the Warburg effect, leads to tumor proliferation and cancer progression. To satisfy their high glycolytic demands, cancer cells have increased NAD^+^ levels and an upregulated NAD^+^ salvage biosynthesis pathway, the main source of NAD^+^ in cells. It is not surprising to find upregulated NAMPT, the rate-limiting enzyme of this pathway, in several human tumors. NAMPT has been proposed as a potent oncogene capable of promoting cell proliferation by increasing the NAD^+^ pool and, consequently, enhancing SIRT1 and PARP1 activation.

NAD^+^ has also been involved in pluripotency and stemness, and through these properties on most human illness including neurodegeneration, immune depletion, and cancer. Indeed, NAMPT and SIRT1 activate OSKM factors and stem signaling pathways, promoting stemness and dedifferentiation from cancer cells to cancer stem-like cells (CSCs). A small subpopulation of CSCs is critical not only for tumor initiation and progression but also for therapy resistance and metastasis. Because of the inefficiency of traditional anticancer therapies, new therapeutic targets for CSCs are being sought. In this context, the NAMPT axis could be a potential new target. However, the inherent toxicity associated with its genetic ablation limits new possible strategies. Partial NAMPT inhibition in combination with other concurrent axis could improve efficacy decreasing toxicity. Differentiation enzymatic or non-enzymatic activities of NAMPT is another strategy to differentiate among different physiological events involved only on cancer. However, the balance is very narrow and must be better defined for therapeutical approaches, since decreasing NAD may lead to a decrease in pluripotency, aging, neurodegeneration, and immune response, while an excessive increase may contribute to cancer. More efforts are necessary to establish the therapeutic window for cancer without contributing to other NAD-derived illness or toxicity.
